# Effect of regional wind circulation and meteorological factors on long-range migration of mustard aphids over indo-gangetic plain

**DOI:** 10.1038/s41598-019-42151-8

**Published:** 2019-04-04

**Authors:** Sayantan Ghosh, Arindam Roy, Abhijit Chatterjee, Samir Ranjan Sikdar

**Affiliations:** 1 Division of Plant Biology, Bose Institute, Kolkata, West Bengal India; 2 Environmental Sciences Section, Bose Institute, Kolkata, West Bengal India

## Abstract

Mustard aphids are a serious problem for *Brassica* oilseed in India causing up to 90% of the crop damage. It was hypothesized that Aphids migrate into the Indo Gangetic plain (IGP) from hilly regions of every year. Exact source and migration pattern of this pest is unknown till date. During their long range migration they infested various places over IGP, which fall on their way of migration. The wind, blown from the hilly regions helps aphids to migrate. Meteorological parameters play a crucial role in this migration of aphids. In this study, we have done the 24 hours air-mass backward trajectory at 100 m above ground level (agl) to detect the source regions of mustard aphids. We have found that mainly Western Himalayan hilly regions act as the source of mustard aphids for IGPs. The dependence upon the micro-meteorological parameters and population dynamics are analyzed and discussed elaborately in this work. In this study, we have proposed the ‘Hop and Fly’ behavior of mustard aphid and further discussed how this migrating behavior could help us to reduce the yield loss of *Brassica*.

## Introduction

Insect migration is the key process by which the population dynamics of many insect pests is being maintained over a vast region^1^. The fascinating migration of Monarch butterfly spending across 3600 km from southern Canada to central Mexico has been well characterized by several studies^2,3^. Different insects take different strategy during migratory flight. Some insects show non-stop flights from the source site during migration (brown plant-hopper) and other migratory insects refuel themselves by taking rest between two consecutive flight^4^. The later behavior is known as multistep migration and is observed in Armyworm, Silver Y moth, and the rice leaf roller^5^. Pest migration has been drawing a lot of interest in recent times.

Aerial insect trapping was the only available method for migration study and this has been used successfully over Indian subcontinent^6^. However, more sophisticated tools have been developed in recent time for tracking insect migration. Use of Doppler and entomological radar and different trajectory based technique have proved useful for studying migratory behavior of insects in various parts of the world^5,7,8^. Some insect pests, like aphids, change their habitats more or less seasonally depending upon the wind track and host availability^9^.

Aphids, members of the order Hemiptera, are one of the most notorious pests for *Brassica* crops in the world^10^. *Lipaphis erysimi* is a serious threat to the cultivation of rapeseed-mustard causing a yield loss of 35–91% per year in India^11^, and till now in *Brassica* germplasm, no resistance is available against this threat^12^. In India, the mustard aphid attacks generally during December and continues till March. Rest of the year, they are literally absent from the plain lands of India. During infection, their population generally builds up on the upper and middle portion of the plant^13^. It was observed the growth stage of host plants may affect the aphid performance. It was reported that the intrinsic rate of increase of the Mustard aphid was higher on host plant during the flowering stage than vegetative stage^14^.

Earlier studies on aphid migration indicate that aphids migrate from hill regions to plain lands of India to avoid lower temperature^15^. In this migratory route, aphids spread their offspring both temporally and spatially, so that, a portion of their next generation can be able to build a seasonally favourable population in near future^6^.

However, the ‘Hills to plain hypothesis’ failed to highlight the proper route of Aphid migration. It also is not able to indicate the exact sources of aphid population and role of migration in population dynamics of aphid.

The present study used a mixed approach of ground base sampling, trajectory based wind analysis and satellite data to explicitly study on mustard Aphid migration over extremely fertile and productive region of Indo-Gangetic Plain (IGP) over Indian subcontinent. The major questions addressed in this the study are:

1. What is the source region of mustard aphid over IGP region?
2. In which direction aphid migrate from source region and what is the major driving force behind this migration?
3. How population dynamics have been affected at different parts of IGP due to differential migration pattern?

## Results

**Figure 1:**
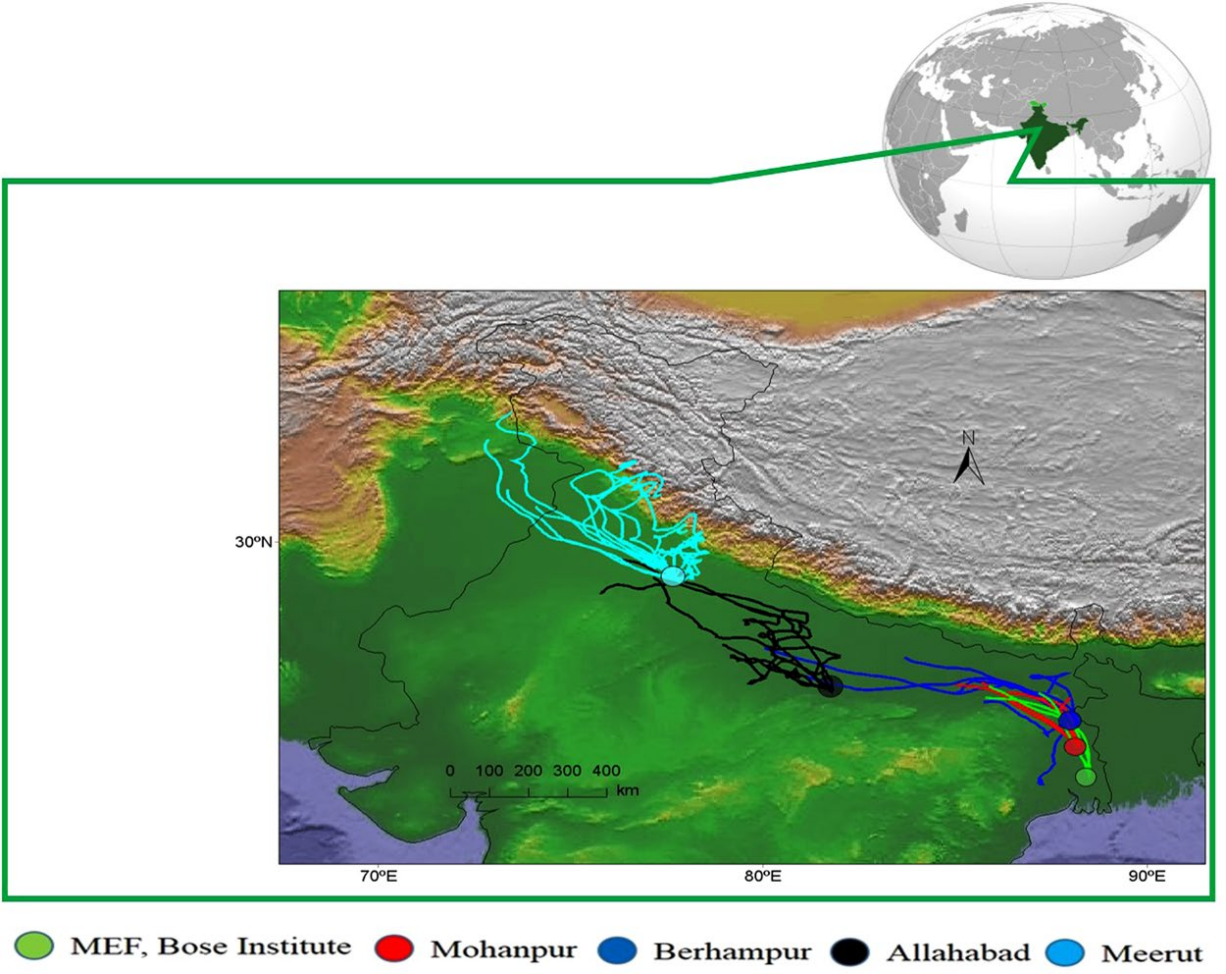
24 h backward air-mass trajectory at 100 m agl over the ground from experimental site, Madhyamgram. This indicated Mohanpur followed by Berhampur, Allahabad and Meerut as the source regions of mustard aphid. This method indicated Western Himalaya as the source of mustard aphids in IGP. (Image source: https://www.ngdc.noaa.gov/mgg/topo/pictures/GLOBALeb3litcolshade_ver1.jpg).

Establishment of aphid population in mustard has been observed in the 2nd week of January during a two year long investigation from 2016–2018 over the study site. Previous studies over the study area indicate infection of aphid has been repeatedly observed in the same week in at least last 5 years. To explain this time dependent infection phenomenon, we have computed 24 hours backward air-mass trajectory at 100 m above the ground from the sampling site (Fig. 1).

The backward air-mass trajectory indicate the source region of aphid population is Mohanpur, an upwind township of Gangetic West Bengal. Literature reviews suggest the aphid infection over Mohanpur is usually observed during the 1st week of January^16^. To investigate further upwind source region, we calculated 24 h backward trajectory from Mohanpur at 100 m agl during the entire 1st week of January. The trajectory indicates the source region for Mohanpur is Berhampur, another township over lower Indo-Gangetic Plain. In a previous study, aerial netting experiment was done at the height of 150 m over Mohanpur and that suggested Berhampur as one of the potential source site of aphid over Mohanpur^6^. The appearance of aphid was observed during the last week of December over Berhampur^17^. Backward trajectories from Berhampur indicate middle IGP as potential source region of aphid and infection of aphid was observed in Allahabaad^18^, a city over middle Indo-Gangetic Plain, during 2nd week of December. Upper IGP is considered as potential source of aphid of middle IGP and it was reported aphid incidence over Meerut, an agricultural region of Indian state of Uttar Pradesh, during early November^19^. Upper IGP is also very close to western Himalayan high altitude eco-region which, according to our hypothesis is the potential source of aphid over Indo-Gangetic regions. Several studies over Western Himalayan regions have been carried out and indicate this as a home for a vast population of Aphids (Supplementary Table 1).

The differential timing of infection of Aphid throughout Indo-Gangetic plain helps us to build a hypothesis of Aphid migration and establishment of infection. We proposed ‘Hop and Fly hypothesis of aphid migration’ on the basis of previous discussion.

1. Aphid remained at nymph stage in the mountains of Himalaya feeding upon their other secondary hosts during rest of the year other than winter. On the onset of winter (November), they start migrating towards plain land regions. Hill to plain migration of aphids has been reported elsewhere^15^. In this study, we postulate that mustard aphid of IGP regions is mainly migrated from hills of western Himalayan regions.
2. Studies across the IGP indicate that aphid infection started in upper IGP during November, in middle IGP during December and in Lower IGP during January. Shifting of initial aphid infection date by several weeks in downwind regions suggested that a population of aphid nymph converted into alate and migrates towards regions with suitable environment for reproduction.
3. In contrast to stations over middle and lower IGP, aphid population dynamics over upper IGP is significantly different. Over upper IGP, aphid population reaches at its peak during fourth week of infestation and the peak sustains there for next 6 week (Fig. 2). Whereas over middle and lower IGP aphid population attains its peak during 9^th^ and 4^th^ week after infestation respectively and readily decrease within a week during1st week of March (Fig. 2). It was noteworthy that the pattern of the peak over middle IGP possesses high similarity with that of lower IGP. Longer peak period over upper IGP as compared to lower and middle IGP might be due to the continuous migration of aphid population from hills to upper IGP region which is situated at the close proximity to the western Himalayan region.

**Figure 2:**
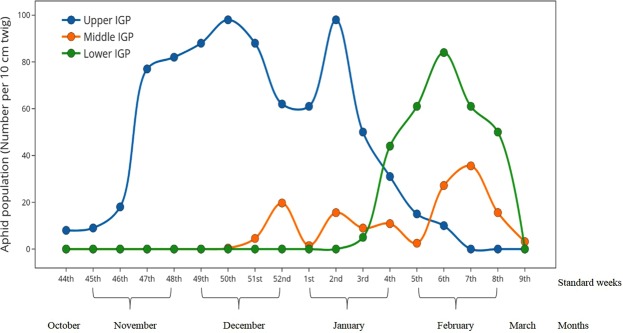
Population dynamics of mustard aphids over different parts of IGP.
4. Temperature over upper IGP region and initial aphid infection suggested that during initial period of infection the average temperature of upper IGP region was around 20 °C (Fig. 3). By the time of mid-December, it falls down to 16 °C which according to our hypothesis is an alarming call for the aphids to migrate. Initial aphid infection over mid-IGP region was observed during December 2nd week when temperature over mid-IGP was observed to be around 20 °C. Temperature over mid-IGP decrease down to around 14–16 °C during December end or beginning of January. During this time aphid infection was observed in lower IGP region^16^.

**Figure 3:**
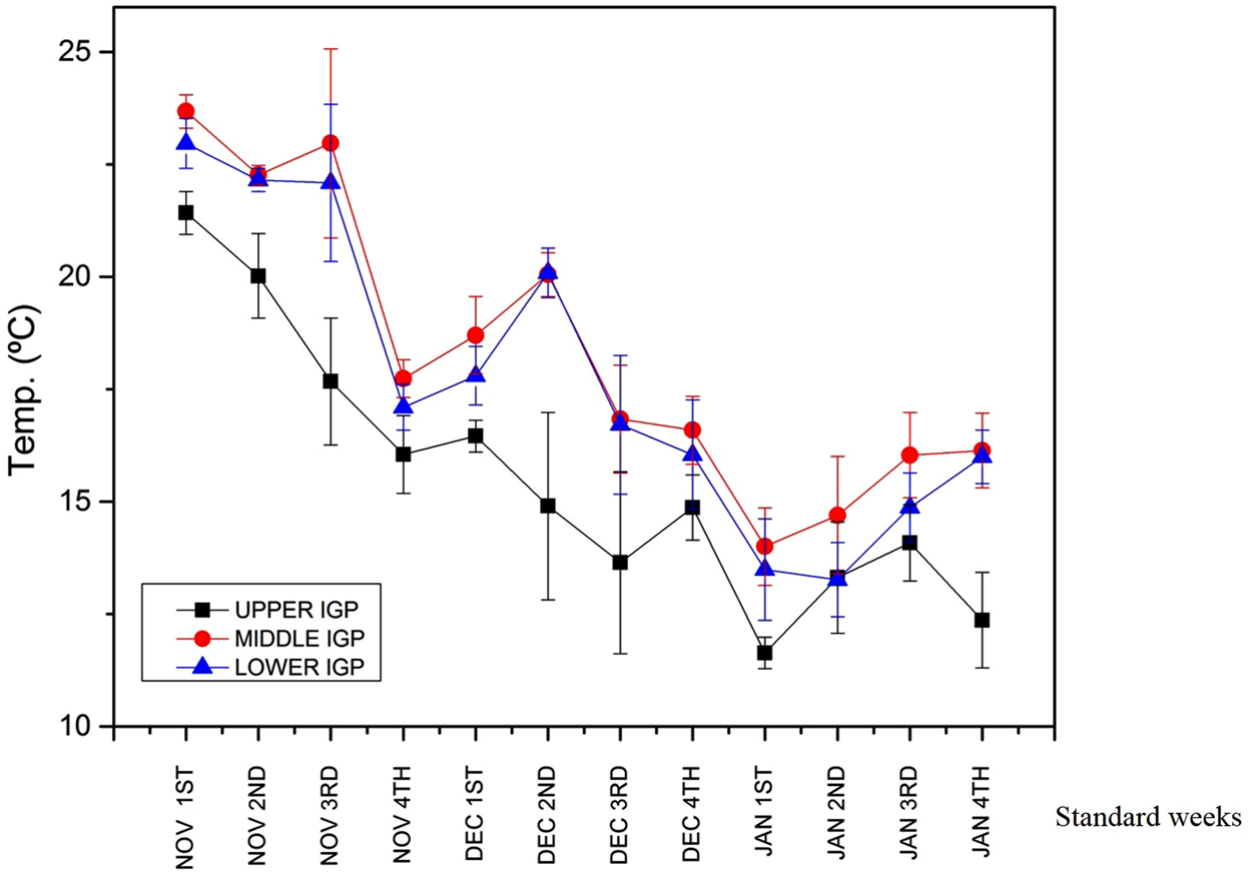
Weekly mean temperature over different parts of IGP.

### Aphid population dynamics with respect to its infestation over IGP

**Figure 4:**
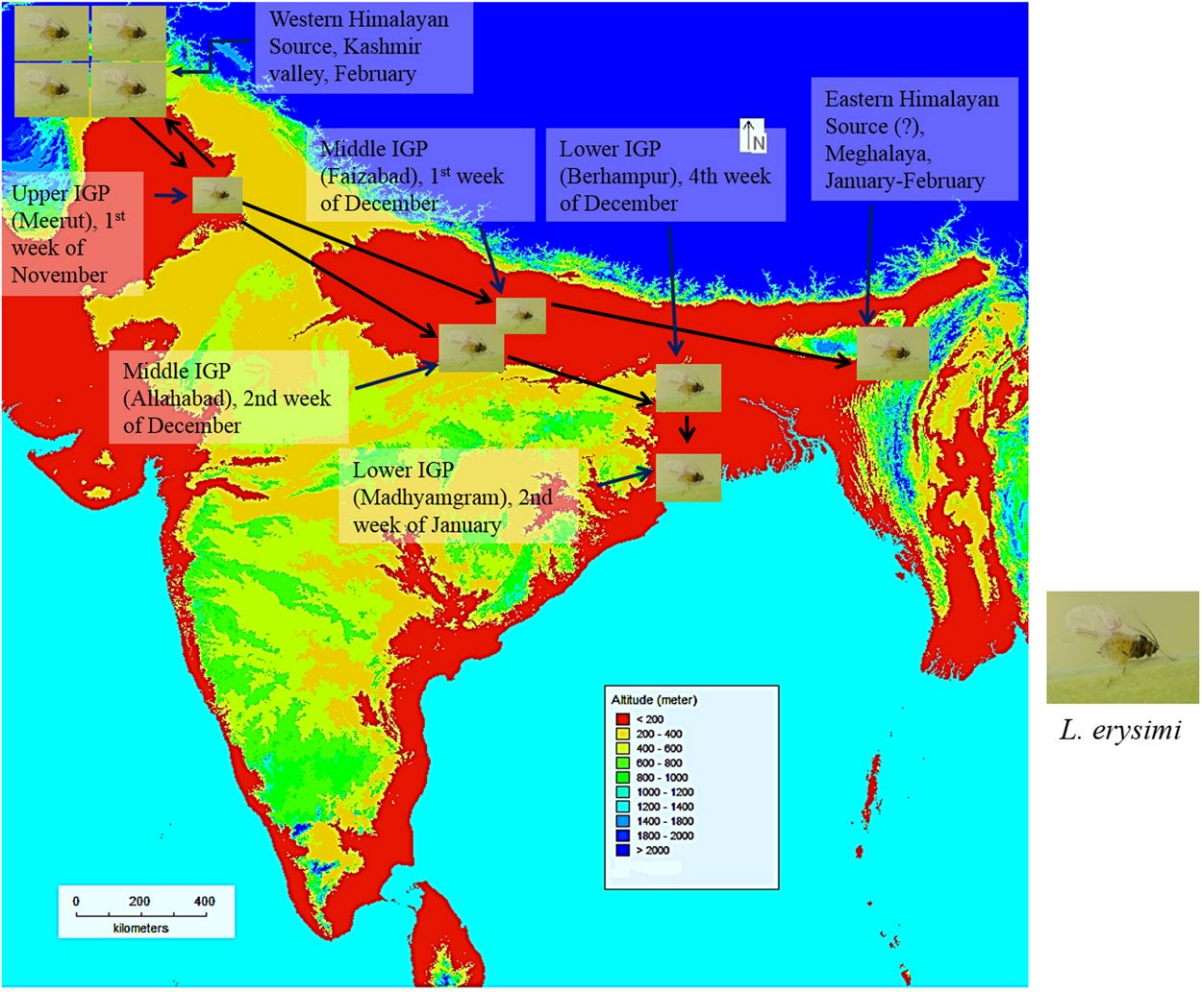
Graphical representation of “Hop and Fly hypothesis”. From Western Himalayan source mustard aphids enter into the upper IGP (Meerut), from there, it fly into middle IGP in two possible ways, one may enter into lower IGP via Allahabad and follows the regular path of their migration over IGP and the second path describes about the migration of mustard aphid in terrai region of Eastern Himalaya, which may act as another source for mustard aphids into Bangladesh and West Bengal. The mustard aphids may go back to their source region at Western Himalaya from upper IGP upon the seasonal change to maintain the population dynamics in Hill regions of Western Himalaya. (Image source: Image of *L. erysimi* was captured by S. Ghosh. Map is produced using elevation data from http://www.diva-gis.org/Data, using DIVA-GIS 7.5 software followed by Microsoft power point modification).

We have taken aphid population dynamics data from different parts of IGP and ensembles into Fig. 4. Meerut is taken as a representative of upper IGP whereas Allahabad and Madhyamgram are used as representative of middle and lower IGP respectively. Aphid population persisted over upper IGP region over a period of 16 weeks whereas it sustains 12 and 6 weeks over middle IGP and lower IGP respectively. The peak population over upper IGP sustains almost for 2 months which in turns helps to establish ‘Hills to plain hypothesis’ of aphid migration firmly. We assume that the hills act as a constant source of aphid population over upper IGP during the entire infection periods. However, during late January the temperature over upper IGP begins to rise continuously which in turn helps to reverse the source-sink relationship between hills and plain. Now the aphid population migrates towards the hill region from upper IGP and waits until next cultivation period.

Population dynamics of over middle and lower IGP found to be almost similar except for the infestation date. In both the areas, aphid population attains its peak during mid-January period (Fig. 2). The aphid population reaches its maxima for a very short period of time over these two regions compared with upper IGP region. This could be explained by similar temperature dynamics over two regions which show similar patterns that could play crucial role in aphid number concentration.

### Aphid infestation through other route over Indian subcontinent

We have also calculated possible aphid infestation pathways throughout various places over Indian subcontinent. Different places with Aphid infestation date and backward wind trajectory have been sown in Supplementary Fig. 1. Aphid infection begins at Gazipur^20^ and Comilla^21^ Bangladesh, during 1st week of January and 3^rd^ week of December respectively, which is also close to the infestation date of our sampling station. Backward trajectory analysis indicates possible sources are lower-IGP and middle IGP. Infection over terrai region of Bengal (Coochbehar) reported during 4^th^ week of December (Supplementary Table 1). Backward air-mass trajectory indicates upper IGP as potential source region.

A study over a high altitude site in Jammu and Kashmir (1500 m agl) reported late appearance of *Lipaphis* in the 2nd week of March which supports our hypothesis^22^. Another study of aphid infection over Pantnagar, Uttarakhand reported aphid infestation starts during December 1st week^23^. Back trajectory analysis indicates Kashmir Himalaya as source region.

During winter months (Dec- Jan), temperature over the high altitude sites over western Himalaya goes down to near freezing level. At that time Aphids migrate towards plain lands of upper IGP regions as described earlier. As the temperature over upper IGP begins to rise during March, those aphids migrate back toward the hill region and infested mustard plants over there. So, we can infer that the aphids not only migrate from the hills to the plain as described by earlier studies but also, follow a ‘to and fro’ movement to avoid the extreme temperature regimes.

## Discussion

A large scale study was conducted on database of museum specimens, to identify both eastern and western Himalaya as a major distribution center of Aphid^24^. According to our study, north-western and Kashmir Himalaya, which is a part of western Himalaya, is acting as the potential source region of *Lipaphis* over IGP. This is a fallacy that although the eastern part of the Himalaya is comparatively closer to the lower and middle IGP, still *Lipaphis* migrating from western Himalaya infected mustard throughout the IGP. The major reason behind this unique phenomenon is the air-mass trajectory that drives the vast population of aphid from western Himalaya during every November towards highly productive crop field of IGP. Thus, this investigation of air-mass trajectory on migrating behavior of a major crop pest provides sufficient evidence for ‘Hills to plain hypothesis’ over Indian subcontinent and pinpoint to the potential source region.

Meteorological factors also play an important role in this migration. Eastern Himalaya receives much more precipitation compared to its counterpart during Indian summer monsoon (June–September)^25^. Aphid population over eastern Himalaya could get easily scavenged and washed away during the entire monsoon season and hence it could not work as potential source region for mustard aphid infection. On the other hand, Western Himalaya receives minimum precipitation during Indian summer monsoon and capable to nourish aphid population for infection during early November.

As previously discussed, temperature works as a rate limiting factor in the whole migration process. Lower temperature is the driving force of the aphid to migrate from the freezing part of western Himalaya to the upper portion of IGP during November. Low temperatures over upper IGP during December helps to initiate aphid migration towards mid and lower part of IGP. This type of temperature dependent pest migration has been studied in different parts of the globe^26,27^. Temperature dynamics also play crucial role in aphid population dynamics over IGP. Longer period of infection is observed in upper part of IGP compare to the middle and lower part of IGP.

Several studies have been published in order to establish aphid forecasting over Indian subcontinent^28,29^. These studies majorly limited in correlating of different meteorological factor of the study site. However, regional air-mass pattern have been totally ignored till date. In this study we have found that the role of meteorological parameters and regional wind pattern are both work simultaneously in aphid establishment as well as its population dynamics. Therefore only a combinatorial model parameterized both meteorological parameters and regional circulation over a vast region of Himalaya and IGP could efficiently predict the aphid infection time over this region.

As discussed in the earlier section, insect migration could occur in both unidirectional or bidirectional manner. Here, one major question to be addressed by us, is ‘whether the studied aphid (*L. erysimi*) does migrate in unidirectional or in bidirectional manner over Indian subcontinent’? The outcome from this study reflects that the migration is mostly unidirectional. Regional wind pattern plays the major role for the migration of this very small insect. The forward wind trajectory from lower IGP during the season of springs (Feb–Mar) indicates air-mass travelling towards Bay of Bengal (Sup Fig. 2). So, the question arises ‘how does the renewal of aphid population occur over the Himalayan source region’? A study over a western Himalayan high altitude site (Chamba) reported first appearance of the Aphid during the second week of February (Sup Table 1). The backward trajectory analysis reveals Chamba receives air-mass from upper IGP region during the time of first aphid infection (Sup Fig. 2). As discussed earlier, *L. erysimi* infection over upper IGP continues till the end of February. This further indicates that this aphid moves back to the western Himalayan hilly region from upper part of IGP during the month of February and reversal of source-sink relationship happens. This concludes a ‘to and fro’ migration of aphids between western Himalaya and upper IGP region whereas, the migration was found to be unidirectional (Hills to plain) for the aphid population moving from western Himalaya to lower IGP. Upper IGP continues to act as source for aphid renewal over the hills of Western Himalaya. After moving back to hills, the aphid further moves toward north and infect the mustard field over Jammu (February)^30^ and Kashmir (March 2^nd^ week) (Sup Table 1).

*Brassica juncea* is an annual plant, cultivated in Rabi season (from September to March) over a vast region of India. It takes about 2 months to become full bloomed plant, which is preferable for aphid attack. In India, there is practice of early mustard cultivation to escape the aphid attack. But this fails often due to lack of proper knowledge of pest management. We observed that, in upper IGP the aphid appears around in the month of November (where the seeds are sown in September), in middle IGP it appears in the month of December (seeds are sown in October) and in lower IGP it appears in the month of January (seeds are sown generally in November). So, we are proposing a reverse cultivation method to avoid crop damage over IGP. If the cultivation starts during October over middle and lower IGP and during December over upper IGP, major crop damage due to aphid infection could be saved. According to our ‘Hop and Fly hypothesis of aphid migration’, they would not able to sit in between their source and lower IGP during November, as, there will be no mustard plant over upper IGP at that time. So, basically, the migration might be hampered due to lack of nutrition source, required for their migration, and thus the crop could be saved.

Although it has been reported multiple times that *Lipaphis erysimi* is a polyphagous pest that fed only on plants of cruciferous family^31,32^ cultivated during the Rabi season. Thereby, there is a probability that the reverse cultivation of only mustard (*Brassica juncea*) might not able to escape the aphid attack completely. The best possible way to escape aphids would be to cultivate all the cruciferous crops in reverse manner. However, to prove this hypothesis, a large scale special study covering multiple sites over IGP is required.

## Methods

The representative stations for the aphid study for present purpose were selected at Meerut (28.9845°N, 77.7064° E) (Upper IGP), Allahabad (25.4358°N, 81.8463°E) (Middle IGP) and Madhyamgram Experimental Farm (MEF), Bose Institute (22.6925°N, 88.4654°E) (Lower IGP). Experiments were done at Lower IGP center, MEF. The dates of appearance of mustard aphids, over IGP were collected from different literatures, as well as it was studied at the experimental field. From the last 10 years’ data, we observed that the appearance of aphids occurs more or less in same time of the year for a particular area. For the study of aphid population dynamics, we trapped a counted number (five) of insects on a non-infected leaf of mustard plant, covered by a muslin pouch. The change in population was monitored till the crop harvest in weekly manner. Inside the pouch, a gradual increase followed by a sharp decrease in aphid population was observed.

24 hours air-mass backward trajectory ending at 100 m above the ground level has been calculated using Hybrid Single Particle Lagrangian Integrated Trajectory Model (HYSPLIT) model over different part of IGP to find out the contribution of long-range transport Aphid migration. National Oceanic and Atmospheric Administration (NOAA) and Australia’s Bureau of Meteorology develop this model. The calculation for this model is a hybrid between Lagrangian approach and eulerian methodology. This particular model can be used in transport, dispersion and deposition of atmospheric pollutants. Only a handful of studies have used models to study insect migration^33,34^. To the best of our knowledge, this study is the first ever to use air-mass trajectory model for insect migration over Indian subcontinent.

The temperature data used in this study has been taken from Modern-Era Retrospective analysis for Research and Applications (MERRA -2) model data over IGP. Area averaged of 2meter daily air temperature (0.5° × 0.625° resolution) was downloaded from Giovanni user interface maintained by NASA.

Maps used here, were taken from the website of National Centers for Environmental Information, NOAA (National Oceanic and Atmospheric Administration). The map is also available in the open source software we used in this study (Meteoinfo) (https://www.ngdc.noaa.gov/mgg/topo/pictures/GLOBALeb3litcolshade_ver1.jpg). The open source map was modified according to requirement using elevation data from http://www.diva gis.org/Data, using DIVA-GIS 7.5 software and further modified in Microsoft power point.

## Supplementary information

Supplementary Informations

## Data Availability

The dataset analyzed in this experiment are available from the corresponding author upon reasonable request.
