# Supplementary material for: Effect of regional wind circulation and meteorological factors on long-range migration of mustard aphids over indo-gangetic plain
Source: Sci Rep. 2019 Apr 4;9:5626. doi: 10.1038/s41598-019-42151-8 (PMC6449332; doi:10.1038/s41598-019-42151-8)

EFFECT OF REGIONAL WIND CIRCULATION AND METEOROLOGICAL FACTORS ON LONG-RANGE MIGRATION OF MUSTARD APHIDS OVER INDO-GANGETIC PLAIN

Sayantan Ghosh [1], Arindam Roy [2], Abhijit Chatterjee [2] & Samir Ranjan Sikdar [1]*.

1. Division of Plant Biology, Bose Institute; 2. Environmental Sciences Section, Bose Institute.

* Corresponding Author.

Sup Table 1: Sowing dates and aphid appearance at different stations over IGP

Serial number	Place	Position	Sowing date	Infestation date	Peak period	References
**1**	Mohanpur	Lower IGP	3rd wk of October	1st wk of January	-	1
**2**	Allahabad	Middle IGP	October	2nd wk of December	3rd wk of February	2
**3**	Meerut	Upper IGP	October	1st wk of November	2nd wk of February	3
**4**	Gazipur, Bangladesh	,,	2nd wk of November,	1st wk of January	1st wk of February	4
**5**	Comilla, Bangladesh	Lower IGP	3rd wk of November,	3rd wk of December	2nd wk of January	5
**6**	Pantnagar	Upper IGP	3rd wk of October	1st wk of December	4th wk of December	6
**7**	Dhiansar, Jammu	Western Himalaya	October	February	-	7
**8**	Coochbihar	Terai	4th wk of November,	4th wk of December	2nd wk of February	8
**9**	Chamba	Western Himalaya	Rabi season	2nd wk of February	2nd wk of March	9
**10**	Faizabad	Middle IGP	4th wk of November	1st wk of December	3rd wk of December	10
**11**	Madhyamgram	Lower IGP	1st wk of November	2nd wk of January	2nd wk of February	-

Supplementary References:

1. Dinda, N. K., Ray, M. & Sarkar, P. Effect of sowing date vis-a-vis variety of rapeseed and Mustard on growth, yield and aphid infestation in Gangetic plains of West Bengal. *The Ecoscan*. **9** (1&2), 21-24, (2015).
2. Dharavat, N., Mehera, B., Nath, S., Patra, S. S. & Rout, S. Effect of Sowing Dates on Population Dynamics of Mustard Aphid (*Lipaphis erysimi*) In Mustard (*Brassica juncea*) Under Allahabad Climatic Condition. *Advances in Life Sciences*. **5** (20), 9109-9113, (2016).
3. Sain, Y., Singh, R. & Kumar, S. Seasonal incidence of cabbage aphid, *Lipaphis erysimi* (*Kalt.*) (Hemiptera: Aphididae) in Meerut region, Uttar Pradesh. *J. Entomol. Zool. Stud*. **5** (6), 314-317, (2017).
4. Biswas, G. C. Comparative effectiveness of neem extracts and Synthetic organic insecticide against mustard aphid. *Bangladesh journal of Agricultural Research*. **38** (2), 181-187, (2013).
5. Sultana, N. A., Khan, M. A. H., Islam, M. N. & Hasanuzzaman, M. Integrated Management of Aphid (*Lipaphis erysimi* Kalt.) In Mustard. *World Journal of Zoology*. **4** (2), 105-108, (2009).

6. Patel, S. & Singh, C. P. Seasonal dynamics of *Lipaphis erysimi* (Kalt.): arrival, peak and migration pattern in tarai region of Uttarakhand. *J. Exp. Zool. India.* **20** (1), 1573-1575, (2017).
7. Sinha, R., Singh, B., Rai, P. K., Kumar, A., Jamwal, S. & Sinha, B. K. Soil fertility management and its impact on mustard aphid *Lipaphis erysimi* (Kaltenbach) Hemiptera: Aphididae. *Cogent Food & Agriculture.* **4**, 1450941 (2018).
8. Chowdhury, S. & Pal, S. Population dynamics of mustard aphid on different *Brassica* cultivars under terai agro-ecological conditions of West Bengal. *The Journal of Plant Protection Sciences*, **1** (1): 83-86, (2009).
9. Verma, S. C., Thakur, D. S. & Kumar, V. Population build-up of mustard aphid (*Lipaphis erysimi* Kalt.) on Mustard (*Brassica juncea* Coss.) under sub humid mid hill zone of Himachal Pradesh, India. *Indian Journal of Hill Farmg*. **15** (2), 30-31, (2002).
10. Bhati, R., Sharma, R. S. & Singh, R. Studies on occurrence of insect-pests of different Brassica species. *Int J Curr Sci*. **14**, 125-132. (2015).

Sup Fig 1: Possible source region of mustard aphid attack over IGP.

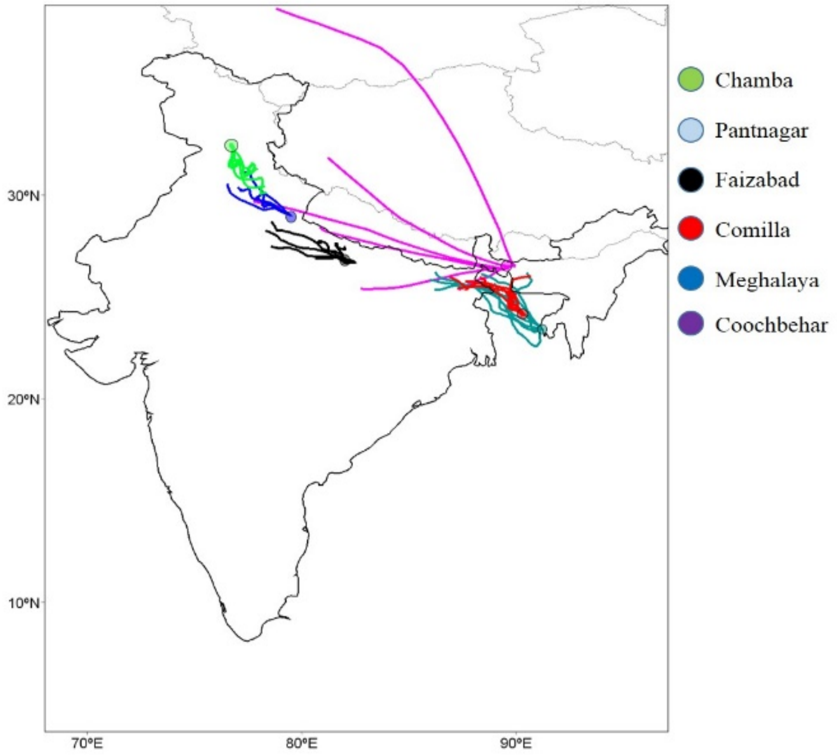

Sup Fig 2: Forward air-mass trajectory at 100m amsl over the ground from experimental site, Madhyamgram shows the air mass flows towards Bay of Bengal. This proves that, mustard aphid migration in lower IGP is unidirectional.

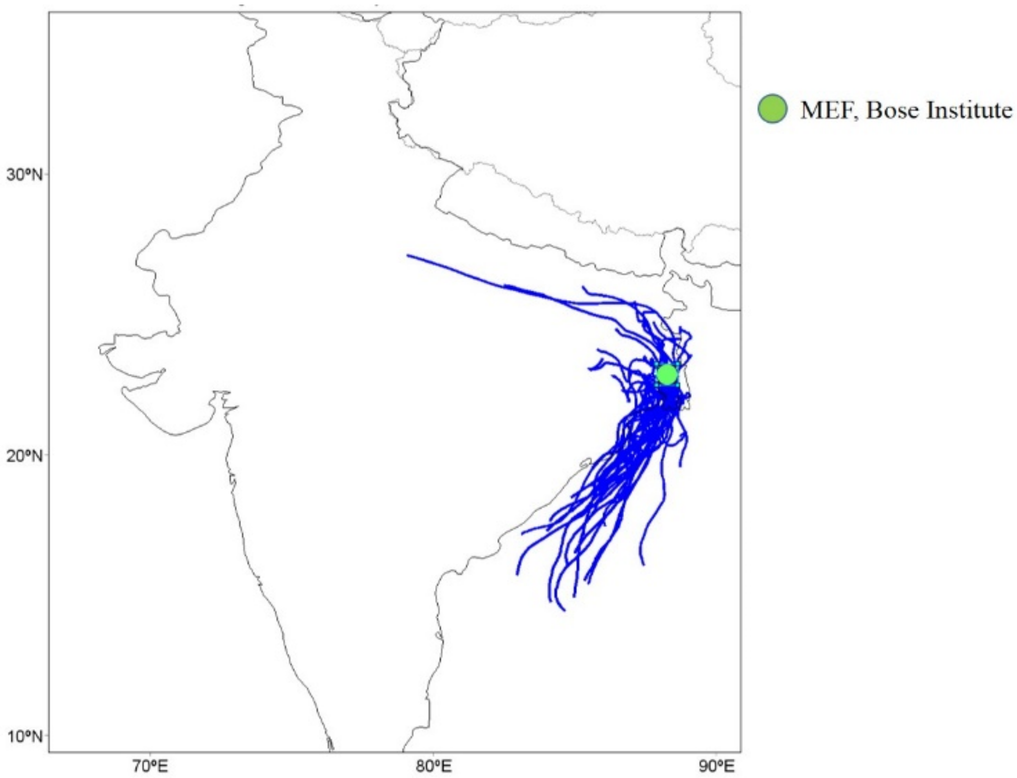

Supplement: Supplementary file 1 — Supplementary Informations [file 41598_2019_42151_MOESM1_ESM.pdf]
